# Prioritizing genes of potential relevance to diseases affected by sex hormones: an example of Myasthenia Gravis

**DOI:** 10.1186/1471-2164-9-481

**Published:** 2008-10-13

**Authors:** Mandeep Kaur, Sebastian Schmeier, Cameron R MacPherson, Oliver Hofmann, Winston A Hide, Stephen Taylor, Nick Willcox, Vladimir B Bajic

**Affiliations:** 1 South African National Bioinformatics Institute, University of the Western Cape, Bellville, Republic of South Africa; 2 Weatherall Institute for Molecular Medicine, University of Oxford, OX3 9DS, UK; 3 Neurosciences Group, Department of Clinical Neurology, University of Oxford, UK

## Abstract

**Background:**

About 5% of western populations are afflicted by autoimmune diseases many of which are affected by sex hormones. Autoimmune diseases are complex and involve many genes. Identifying these disease-associated genes contributes to development of more effective therapies. Also, association studies frequently imply genomic regions that contain disease-associated genes but fall short of pinpointing these genes. The identification of disease-associated genes has always been challenging and to date there is no universal and effective method developed.

**Results:**

We have developed a method to prioritize disease-associated genes for diseases affected strongly by sex hormones. Our method uses various types of information available for the genes, but no information that directly links genes with the disease. It generates a score for each of the considered genes and ranks genes based on that score. We illustrate our method on early-onset myasthenia gravis (MG) using genes potentially controlled by estrogen and localized in a genomic segment (which contains the MHC and surrounding region) strongly associated with MG. Based on the considered genomic segment 283 genes are ranked for their relevance to MG and responsiveness to estrogen. The top three ranked genes, HLA-G, TAP2 and HLA-DRB1, are implicated in autoimmune diseases, while TAP2 is associated with SNPs characteristic for MG. Within the top 35 prioritized genes our method identifies 90% of the 10 already known MG-associated genes from the considered region without using any information that directly links genes to MG. Among the top eight genes we identified HLA-G and TUBB as new candidates. We show that our *ab-initio *approach outperforms the other methods for prioritizing disease-associated genes.

**Conclusion:**

We have developed a method to prioritize disease-associated genes under the potential control of sex hormones. We demonstrate the success of this method by prioritizing the genes localized in the MHC and surrounding region and evaluating the role of these genes as potential candidates for estrogen control as well as MG. We show that our method outperforms the other methods. The method has a potential to be adapted to prioritize genes relevant to other diseases.

## Background

**Figure 1:**
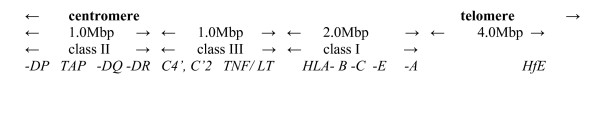
A simplified map of extended MHC.

Collectively, ~5% of western populations are afflicted by autoimmune diseases, such as type I diabetes, rheumatoid arthritis and multiple sclerosis (MS) [1,2]. Hormonal influences are strongly implicated by their frequent female bias and onset between puberty and the menopause [3]. The strongest known susceptibility loci mostly reside in the major histocompatibility complex (MHC) [4], as in MS, where the very tightly linked HLA-DR51 and -DR15 predispose significantly [5]; there may also be an independent association with the loosely linked HLA-A3 [6] or nearby loci (A simple map of extended MHC region is provided in Figure 1). Most workers agree that mainly T cells specific for myelin antigens mediate the damage, though autoantibodies may also contribute in some patients.

There are additional clues to pathogenesis in the particularly well defined subgroup of European patients with early-onset myasthenia gravis MG (EOMG). Their muscle weakness is clearly mediated by highly mutated, high affinity, complement-activating, IgG antibodies to the acetylcholine receptor (AChR). EOMG patients show a 3:1 female bias and onset almost always after puberty but before age 45 years. They subside to below control frequencies in patients with onset after age 50 [7]. The HLA-associations with -DR3 and especially -B8 in the class I region extend at least a further 1 Mbp telomerically towards HLA-E – the 'target region' for EOMG – though they decline nearer to HLA-A, and centromerically towards -DR [7]. Some reports map the predisposing 'MYAS-1 gene' to the class III region near TNF [8]. In the UK, these associations appeared even stronger in the females than the males [7].

In the normal thymus, some epithelial cells express AChR subunits and HLA-DR; presumably, their proper function is to induce self-tolerance in newly developing T cells [9]. However, in EOMG, these cells appear 'hyperplastic' and may be responsible for autoimmunizing helper T cells [10,11], and for provoking the characteristic lymph node-type infiltrates, eg by releasing chemokines [12,13]. Rare myoid cells express the complete AChR, and are implicated in formation of the characteristic germinal centers (GC) nearby, many of which are AChR-specific [14]. It is probably in these sites of Ig class switching and somatic hypermutation [15] that the primordial autoantibodies evolve and diversify so that, like those in most patients' sera, they recognize the native conformation of the intact receptor. This evolution can apparently stop at intermediate stages with autoantibodies exclusively of low affinity that are hard to detect in standard assays ('seronegative') but are nonetheless pathogenic [16] and associate with mild thymic hyperplasia [17].

Evidently, there are many stages at which susceptibility genes could contribute, including those mentioned above. Others may include the 'danger signals' and/or cytokines [18] that favor initial T cell autoimmunization rather than tolerance, the strength of the primordial antibody response and of the consequent complement attack on myoid and epithelial cells [17,19] which may depend on complement receptors and regulators as well as activated components, and may also amplify the infiltration. Furthermore, other genes may influence the terminal effector mechanisms at the motor endplates in muscle, or protect against them, for example, via complement regulators or rapsyn over-expression [20], though there are no clinical hints of hormone involvement at this stage.

Hormonal influences also seem likely in systemic lupus erythematosus (SLE) and Graves' disease, which again show female biases and onset in young adults. They may be especially relevant to MG as both are mediated by autoantibodies and both show associations with HLA-DR3 [21,22]; complement is also heavily involved in SLE. Indeed, in animal models of SLE, gonadectomy clearly has major effects on susceptibility/resistance [3,23]. In humans, SLE is well known to 'flare' during pregnancy, when estrogens and progesterone levels are highest [3] and there is a bias in favor of Th2 responses. There may also be exacerbations in MG during pregnancy or menstruation [24,25]. In addition, reduced testosterone could also be important in auto-immune diseases [26].

In general, steroid hormones are recognized initially by intracellular receptors that then bind to Hormone-Response Elements on DNA and control the expression of some of their target genes. Macrophages, T and B cells also express estrogen receptors (ERs) [27]. Indeed, estrogens regulate the expression of genes that function in macrophage activation and cholesterol homeostasis [28]. Another link between the endocrine and immune systems is that ERs are involved in the development of the thymus, which is smaller in ER-alpha knockout mice [29]. Interestingly too, expression of ER-alpha is higher in MG than control thymuses. While that could be an effect of the autoimmune response, deregulated ER expression could instead contribute to its induction, maintenance and progression [30].

We have adopted an independent approach for locating susceptibility genes that are potentially: a/estrogen responsive and b/involved in MG. Our method has been compared with several other computational methods for prioritizing disease-associated genes, and on the example of MG we have demonstrated that our results outperform the others. For 283 candidate genes from the extended MHC region flanked on both sides, we looked for the estrogen-response elements (EREs), as well as for other information that either show that genes are estrogen-responsive or differentially expressed in the EOMG. We combined that with other relevant information from the existing gene annotation and produced a score for each gene. Eventually, based on that score, we were able to rank genes for their responsiveness to estrogen and relevance to MG and among top eight genes we identified HLA-G and TUBB as new candidates. Our method has a potential to be generalized for other diseases and other sex hormones, which is important as many diseases are affected by hormones. This appears to be a pragmatic and inexpensive strategy to prioritize candidate genes for experimental investigation of potential involvement in a disease. Consequently, our results open up possibilities for experimentalists to focus on a small number of strong gene candidates for further experimental studies.

## Results and discussion

We have developed a computational method to prioritize genes from a genomic region that are both responsive to estrogen and involved in a disease (see Materials and Methods). Our method uses various types of gene information (such as responsiveness to estrogen, functional annotation, etc.) and combines them into a scoring scheme that allows for genes prioritization. We have demonstrated the method on the example of MG where the top ranked genes from the prioritized list are enriched with genes already known to be associated with MG. We also compared our method with the other available methods and have shown that it outperforms the others.

### (a) Screening for potential estrogen responsiveness

Among the 283 genes in the target genomic HLA-region, our screening identified 96 genes with predicted EREs. We found numerous potentially estrogen-responsive HLA-region genes in the various databases we searched (Additional file 1, Supplementary Table S1). Notably, they include *HLA-A, -G, -E, and -B*, in the class I region, -*DRB1, TAP2 *in class II, *TNF *and *LTA *(lymphotoxin) – which are both very close to *MYAS 1*- and *HSPA1A *and *B, C2 *and *CFB *complement factors in class III; also *TUBB *(close to HLA-E, a microtubule protein that recurs in several independent screens).

These genes were then weighted and ranked as described in (viii, Materials and Methods) (See  Additional file 1, Supplementary Table S1). Table 1 highlights the 25 ERE-containing HLA region genes with ranks in the top 50 prioritized genes; the estrogen responsiveness of 6 of these genes has been verified experimentally (see See Additional file 1, 'KBERG column' of Supplementary Table S1). Clearly, therefore, numerous HLA-region genes are estrogen-responsive.

**Table 1:** Potential estrogen-responsive genes in the HLA region

**Estrogen-responsiveness identified by**	**All 283 genes**	**Genes in top 50 ranks**
our own screening for EREs	96	25

KBERG data base	11	6

**ERTargetDB**:-		

ChIP-on-chip-confirmed	1	0

microarray evidence	4	3

predicted EREs	31	12

Scanning the eVOC ontologies for the 283 HLA-region genes, we found 157 that are expressed in normal muscle and 104 in normal thymus (See Additional file 1, Supplementary Table S1). At most, MG only involves minority components of each of these tissues.

Interestingly, our parallel screening of a list of 59 reportedly MG-associated genes (See Additional file 1, Supplementary Table S2, see column predicted EREs) detected EREs in the gene *CCR2, IFN-γ, IL-6 *and *IL-7 *but in none of the other chemokines or cytokines listed, nor in a range of co-stimulatory molecules including *CTLA-4*, nor in *PTPN22*, a negative signaling molecule with many autoimmune associations [31]. Since this study focuses on HLA-region genes, only those of the 59 MG-associated genes that reside in this region will be analyzed further.

Ten genes out of 59 MG-associated genes are localized within 10.8 Mbp region we analyzed (See Additional file 1, Supplementary Table S2, highlighted in Yellow). Nine of these genes were ranked among the top 35 ones (the p-value of enrichment after Bonferroni correction for multiplicity testing is 9.99 × 10^-13^, with multiplicity correction factor 283; we used right-hand side exact Fisher's test based on hypergeometric distribution). The tenth MG-associated genes, MYAS-1, does not have the exact genomic coordinates and thus was not suitable for this analysis, so we did not consider it. The remaining nine genes, HLA-DRB1, HLA-DPB1, HLA-B, HLA-A, HLA-DQB1, HLA-DQA1, TNF, HLA-DRA and LTA had rank positions 3, 5, 9, 16, 20, 24, 27, 30 and 35 respectively. This suggests that our ranking method seems meaningful as it can identify 90% of the 10 already known MG-associated genes from the considered region among the top 12.5% (35/283) prioritized genes without using any information that directly links genes to MG (1 MG related gene in the top 1.06%, 2 MG related genes in the top 1.76%, 3 MG related genes in the top 3.18%, 4 in top 5.65%, 5 in top 7.06%, 6 in top 8.48%, 7 in top 9.54%, 8 in top 10.60% and 9 in top 12.36%).

### (b) Cross-checking against genes differentially expressed in the MG thymus

We checked two datasets to identify any HLA-region genes reported to be differentially expressed in the thymus of female MG patients by [12,13]. The former include *HLA-C*, -*DQA*, -*DQB *and -*DOB*, and *C2*, and the latter *TNF *and *CFB *too; also – in the hyperplastic sub-groups -* HLA-B*, -*DQA*, and -*DPA *as well as *TUBB *(Additional file 1, see details in Supplementary Table S1).

### (c) Functional properties of the top 50 prioritized genes

The top 50 genes were next analyzed in more detail according to their functional properties using GO annotations and the DAVID tool [32]. The functional annotation and the pathways mapped by top 50 genes are presented in Additional file 1, Supplementary Table S3.

### (d) Comparison with other available methods

**Figure 2:**
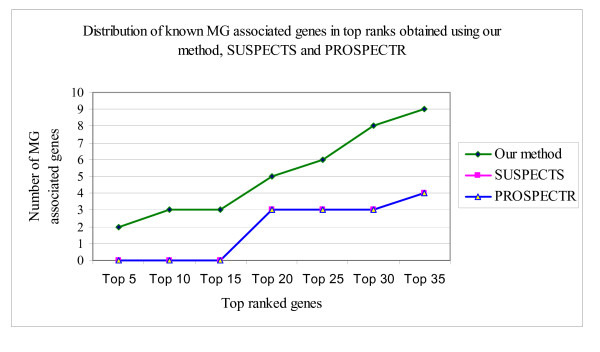
Comparison of results obtained using our method, SUSPECTS and PROSPECTR.

We explored possibility to use other reported methods [33-40] for prioritizing disease-associated genes in the context of our study. In comparison with the existing methods that were available for the analysis, our method was able to identify a greater number of known MG-associated genes in the top ranks (Figure 2).

We want to point out that the set of features we have selected does not determine uniquely Myasthenia Gravis (MG) associated genes. They, however, indirectly characterize MG and our assumption of relevance of estrogen in MG. Indeed, all gene prioritizing methods use various sets of features to indirectly characterize a disease, but none of these feature sets is sufficient to characterize the disease completely. For this very reason, it is not useful to search for the presence of these features for all genes in the human genome. The rational way to utilize feature information is to analyze genes located in the genomic segments that are shown to contain disease-associated genes which in our study are MG-associated genes. Such segments are usually obtained by the linkage analysis. In such a case, one can make a rational assumption that genes from these segments are enriched with (in our case) the MG-associated genes, and thus ranking genes for relevance to MG based on the selected features will be more efficient and more meaningful. As our results show, the already known MG-associated genes from the MHC and surrounding region are located among the top ranked genes. Moreover, the selection of the MHC and surrounding region for the search for MG-associated genes was guided by the linkage analysis results that suggest that the epicenter of the early-onset MG is within MHC.

The methodology mentioned above and described in Materials and Methods has enabled us to identify and prioritize genes likely to be responsive to estrogens and/or potentially MG-associated. We ranked all known genes localized on the 10.8 Mbp region of chromosome 6 based upon predicted EREs, genes' differential expression in MG thymi (which are likely sites of autoimmunization), their known responsiveness to estrogen, and their annotated biological roles. Nine of our 35 top prioritized genes already feature in a set of 59 known MG-associated genes (See Additional file 1, Supplementary Table S2); it is important to note that only 10 of these 59 map within the 10.8 Mbp region we analyzed (See Additional file 1, Supplementary Table S2, highlighted in Yellow); the p-value of enrichment for known MG-associated genes after Bonferroni correction for multiplicity testing is 9.99 × 10^-13 ^(using a multiplicity correction factor 283, and right-hand side Fisher's exact test based on hypergeometric distribution). These nine MG-associated genes include several that have been strongly implicated since the 1970s – 1990s, notably *HLA-DRB1 *(rank 3), *HLA-B *(rank 9), *HLA-DQB1 *(rank 20), *TNF *(rank 24) and *HLA-DQA1 *(rank 27). This prioritization of several already known MG-associated genes strongly supports the validity of our ranking method. Moreover, out of 283 genes, 99 (See Additional file 1, Supplementary Table S4) are localized between DR3--B--A1 (genomic location 30,017,016–32,572,245). Of these 99, 24 are ranked in the top 50; 22 from top 50 reside between DR in class II and HLA-E in class I (30,565,198 – 32,572,245) where associations seem strongest in MG (See Additional file 1, Supplementary Table S5).

In order to show that our selection of weights used for the five groups of features has no resemblance to the set of features that produces the best ranking of the MG-associated genes, we compared the scores and ranks of the MG-associated genes obtained using weights described in the Methods section with those obtained using the optimized weights. To obtain the optimized weights for the five groups of features we used we systematically changed the weights within the range from 1 to 10 if the feature was present, and we set the weight to 0 if it was absent. For each combination of the weights, we made the ranking of all genes from the analyzed region. At the end, we selected the best set of weights (optimized weights) as those that place all nine known MG-associated genes in the smallest set of the top ranked ones. We obtained in this way a set of weights that places all these nine genes in the top 30 ranked genes, as opposed to the top 35 genes that we obtained by our selection of weights. The comparison between our originally selected set of weights and the optimized set are provided in Additional file 1, Supplementary Table S6. This comparison shows that the original weights have no resemblance to the optimized ones, thus proving that we did not specifically tuned original weights to achieve good ranking of the MG-associated genes. The problem that appears with the optimized weights in Additional file 1, Supplementary Table S6 is that it is difficult to provide suitable interpretation of these weights.

We attempted to compare several of the reported methods [33-40] for prioritizing disease-associated genes. Some of these methods, such as [35,38] are not available as web-tools, so we were not able to use them. The scope of the available tools is limited by the data resources and technologies they use, such as gene ontologies, sequence similarities, and/or expression. For example, SUSPECTS require list of genes already known to be involved in the disease under investigation as a yardstick for comparing and ranking 'new' genes. Unlike these, our disease gene prioritizing method: a/does not require prior information about disease associated genes, and b/is based fully on an *ab initio *approach to prioritize candidate disease genes, which makes it unique and likely applicable to many diseases affected by estrogen or other sex hormones. Only two (SUSPECTS and PROSPECTR) of the six methods that have available web-tools for prioritizing disease-associated genes, produced any results for MG. As can be seen from Figure 2, our method significantly outperforms both of these.

The top ranked genes from the considered region include almost all MG-associated genes from the region. We therefore propose that the top 50 genes in the prioritized list have high probabilities of involvement in MG and also of estrogen-responsiveness. Indeed, the latter has been experimentally verified for two of these. *HLA-DRB1 *(rank 3) is already strongly implicated in MG susceptibility, though DRB3 may be even more so [41]. The other is *TUBB *(rank 8), a microtubule protein; although it may seem an unlikely candidate, it may warrant further experimental evaluation because axon guidance molecules have recently proved to be strikingly expressed by GC B-cells [42]. The top ranked genes are involved in pathways such as antigen presentation, type I diabetes, cell adhesion and natural killer cell-mediated pathways (See Additional file 1, Supplementary Table S3). Approximately 90% of these genes are involved in the immune response and ~79% are involved in antigen processing and antigen presentation (See Additional file 1, Supplementary Table S3); moreover, some of these genes have been implicated in autoimmune diseases like diabetes mellitus, rheumatoid arthritis and MG. The details of few top ranked genes and their relevance to autoimmune diseases and MG have been provided in Additional file 2, supplementary material M1.

## Conclusion

We have developed and successfully implemented a computational method to prioritize genes relevant to MG in a strongly MG-associating region on chromosome 6. The methodology we proposed is sound, as demonstrated in two ways: a/in the top 35 prioritized genes (12.5% of all analyzed genes) there are 90% (9 out of 10) already known MG-associated genes from the analyzed genomic region; b/in comparison with the other available methods that were available as public tools, our methods performs considerably better.

We are aware that our study is limited to one particular region of chromosome 6 and that other genes beyond this region and on other chromosomes could also be involved in MG. In addition, estrogen receptors (ER) can also affect expression of other genes by forming protein-protein complexes with other transcription factors such as activator protein-1 (AP-1), Sp1 family, nerve factor-β (NF-β), which in turn can bind to gene promoters and regulate their expression, indirectly broadening the range of estrogen-responsive genes. Therefore, the EREs, though good indicators, only partially reflect a gene's potential for responsiveness to estrogens. Also, our study is exploratory in nature, providing information that can be helpful for further follow-up experiments. It could be deepened and enhanced in many ways, but our primary aim was to prioritize genes based on potential links between MG and estrogen by a meaningful ranking method, thus opening avenues for more sophisticated and detailed future studies. Any data specifically on effects of estrogen in MG patients would obviously be an invaluable bonus. Hormonal influences on expression of susceptibility genes might well contribute to the gender biases and/or early-onset in autoimmune diseases. Moreover, we believe that our method could be adapted to prioritize genes relevant to other diseases whose susceptibility to hormonal influences is frequently unjustly neglected. In that case, however, in our opinion the same weights cannot be used as the weights are disease specific. However, we think that the same methodological steps could be applied for diseases other than MG. Determination of the weights could be objective and data driven, i.e. computational, as we have used to determine the optimized weights. However, these may happen to lack suitable interpretation, as we have experienced exactly in the case of MG.

## Methods

### Overview of our methodology

(i) We first identified 283 genes in the MHC region, and (ii) searched computationally for EREs within them [43]. We then checked against two databases of documented estrogen-responsive genes, namely (iii) KBERG [44] and (iv) ERTargetDB [45], neither of which is comprehensive. We further weighted all genes from the analyzed region according to (v) their expression in the thymus in MG subgroups; (vi) functional annotation using gene ontology (GO), and (vii) eVOC ontologies. Finally, all genes were ranked (viii) according to a combined overall score: the top 50 genes were cross-checked (ix) against a set of reportedly MG-associated genes, and also subjected to GO analysis for inspection of their main functional categories [46]. The sensitivity of the method, to identify the known genes already associated with MG in top ranked positions, was compared with other available methods (x). It should be noted that no information about MG is contained in the GO descriptions we used.

#### (i) Extraction of 10.8 Mbp region

The extended MHC is a 7.6 Mbp stretch in the 6p22.2-p21.32 region of chromosome 6 [47]. We also included upstream and downstream flanking regions, thus extracting a total of 10.8 Mbp from 6p22.3-p21.31 (chromosomal localization 23500000–34300000) region using UCSC Browser  based on genome build hg18 (NCBI build 36.1). Using UCSC browser, we extracted a list of 805 UCSC IDs that correspond to 283 gene symbols (Supplementary Table S1) for known genes localized in this region, together with their chromosomal coordinates. The region, 30,017,016–32,665,603 contains two haplotypes; DR3--B8--A1 and DR15--B7--A3 and former has been associated with EOMG. Further within this region we have a sub-region extending from DR- in class II to HLA-E in class I localized between co-ordinates 30,565,198 – 32,572,245, where associations seem to be strongest for MG.

#### (ii) Prediction of Estrogen Response Elements (EREs)

The tool Dragon ERE Finder version 2.0 [43] was used to predict the EREs on the whole stretch of 10.8 Mbp. That predicted 470 EREs on both strands in [-3000,+200] promoter regions at the 5'ends of genes.

#### (iii) Matching to genes from KBERG database

The genes from the target MHC region were cross-checked against all the 1516 experimentally confirmed estrogen-responsive genes in the KBERG database [44].

#### (iv) Matching to genes in ERTargetDB database

The current version of ERTargetDB [45],  contains:- (a) 40 genes with 48 experimentally verified ERE direct binding sites and 11 experimentally verified ERE tethering sites; (b) 42 genes identified via ChIP-on-chip assay for estrogen binding (c) 355 genes from gene expression microarrays; (d) 2659 computationally predicted genes.

#### (v) Matching to genes in published microarray datasets derived from MG thymi

We next cross-checked the 283 MHC genes against two datasets of genes differentially expressed in MG thymi; one [13] was downloaded from Array Express  and another that was provided as supplementary material with the publication [12]:-

a) [12] used microarray technology to identify novel molecules potentially involved in MG pathogenesis. They generated lists of 157 up-regulated and 227 down-regulated genes in hyperplastic thymus samples from 45 female MG patients relative to 33 normal females.

b) [13] performed microarray analyses to identify genes differentially expressed in thymic samples from females in different MG subgroups versus non-myasthenic female controls. Thymic samples were collected from: 1) four non-MG adult controls (15–19 years old); 2) four (19–25 year old) seropositive MG patients with mild thymic hyperplasia (with ≤ 2 GCs per section); 3) five (18–22 year old) seropositive MG patients with marked thymic hyperplasia (≥ 3 GCs per section), 4) three (16–22 years old) seronegative MG patients (with few GCs if any). This data was downloaded from Array Express and was analyzed by centralizing the data at mean = 0 and SD = 1 and calculating the fold change in expression of genes in diseased states as compared to controls. The top 5% up- and down-regulated genes were used for further analysis.

#### (vi) GO analysis

GO [46] term analysis for all the genes in Additional file 1, Supplementary Table S1 was performed using DAVID (The Database for Annotation, Visualization and Integrated Discovery) version 2.0 [32].

#### (viii) eVOC ontologies

The eVOC ontologies [48,49] allowed us to assess the differential expression of genes in muscle or thymus based on EST, SAGE and microarray data. A public version of eVOC is available at .

#### (viii) Gene scoring and ranking

We list information collected for all 283 genes in Additional file 1, Supplementary Table S1. In total, we used 30 different types of information (features) that characterize each of the genes in terms of its potential to be controlled by estrogen as well as its potential to be MG-associated. However, these features do not imply the same level of confidence – for example in their estrogen-responsiveness or involvement in autoimmune responses/diseases. Thus some genes are already known to be estrogen-responsive from experimental data (KBERG data); others merely appear to have an ERE in their extended gene loci. Furthermore, the existing GO annotation implicates many genes in autoimmune responses, antigen presentation and other immunological functions. Thus, to integrate all this information into a meaningful score we somewhat arbitrary assigned weights to the available features, with scores ranging from 0 to 10, based on the following logic. The scores assigned were: (1) genes in KBERG were assigned weight 10; (2) all GO categories were assigned weight 8 (these are the functional categories assigned based on multiple experimental evidences); (3) ERE predictions (our method and ERTargetDB) and presence in ChIP-on-CHIP data in ERTargetDB were assigned weight 8 (more relevant for estrogen response in general and not specific for MG); (4) microarray evidence was assigned weight 5 (since the genes have been implicated in MG pathogenesis – though not specifically); and (5) eVOC categories were assigned a score of 1 (tissue types generally relevant for MG but not specific enough to provide higher weight). If the feature considered has not been known to be associated with the gene, we assigned it the weight of 0.

Finally, we summed all weights assigned to all features for each of the 283 genes and ranked all genes based on that score. The higher the score, we expect that the gene is more likely to be controlled by estrogen and be MG-associated (Additional file 1, Supplementary Table S1).

#### (ix) Matching known genes to those associated with MG

A list of 72 MG-associated genes was retrieved from gene cards [50], Version 2.37 (Sept 23, 2007). These genes were manually checked for their relevance to MG and we finally obtained a set of 59 such genes (Additional file 1, Supplementary Table S2).

#### (x) Comparison of our method with other published methods

This study is performed based on the new methodology that we introduced for ranking disease related genes. As there are several methods/tools developed for this purpose, such as GeneSeeker [33], Disease Gene Prediction (DGP) [34], SUSPECTS  and PROSPECTR [35,37], G2D [36], POCUS [38] and methods published by [39,40]. We compared the quality of the resulting prioritized gene lists for relevance to MG by our own and these other methods. Only two tools; SUSPECTS and PROSPECTR were able to produce ranked lists of candidate genes from the target region and these results in comparison with ours are given in Figure 2.

## Abbreviations

MHC: Major histocompatibility complex; MG: myasthenia gravis; EOMG: early onset myasthenia gravis; EREs: estrogen response elements.

## Authors' contributions

NW initially proposed the study. MK, VBB and NW conceptualized the study, analyzed results and wrote the manuscript. MK and VBB performed the analysis. SS, CRMP, OH, WAH provided data and analyzed results. ST analyzed results.

## Supplementary Material

Additional File 1 **Supplementary Tables – Supplementary tables and lists supporting the analysis**. These are the supplementary tables provided in the excel format with .xls extension. Click here for file

Additional File 2 **Supplementary material M1- Descriptions of a few top ranked genes**. This file provides the details of a few top ranked genes summarizing the role of these genes in autoimmune diseases and emphasizing on the importance of these genes as potential targets for MG. Click here for file
