# Supplementary material for: Prioritizing genes of potential relevance to diseases affected by sex hormones: an example of Myasthenia Gravis
Source: BMC Genomics. 2008 Oct 13;9:481. doi: 10.1186/1471-2164-9-481 (PMC2592250; doi:10.1186/1471-2164-9-481)
Supplement: Additional File 2 — Supplementary material M1- Descriptions of a few top ranked genes. This file provides the details of a few top ranked genes summarizing the role of these genes in autoimmune diseases and emphasizing on the importance of these genes as potential targets for MG. [file 1471-2164-9-481-S1.pdf]

**Human leukocyte antigen HLA-E and HLA-G** molecules act as powerful modulators of the innate immune response. HLA-G is a non-classical HLA class I molecule, which was initially shown to protect the fetus from mother's immune system. HLA-G is ranked at position one in the analysis presented here and has been demonstrated to play an immunomodulatory role in MS through Fas/FasL-mediated mechanisms [1]. HLA-G has been found to be expressed in lymphocytic leukemia patients as compared to the healthy controls [2]. In esophageal squamous cell carcinoma the expression of HLA-G is a characteristic feature and may be a potentially useful prognostic indicator [3]. The polymorphisms in HLA-E and HLA-G can independently and synergistically influence susceptibility to heterosexual acquisition of HIV-1 [4]. HLA-E is also involved in host response to pathogens, as shown for bacterial infections in sickle cell anemia [5]. HLA-E is ranked 10 in the present analysis; [6] reported that a nearby marker was close to one edge of the plateau of maximum susceptibility in EOMG. Interestingly, the first reported autoimmune association with HLA-E itself was with type I diabetes, and showed striking interactions with onset-age [7].

**TAP2** is a member of the superfamily of ATP-binding cassette (ABC) transporters and is ranked at position two in our analysis. The protein encoded by this gene is involved in transport of peptides from the cytosol to the endoplasmic reticulum where they can be loaded into HLA-class I molecules for subsequent presentation to $CD8^+$ T cells. It is interesting to note that TAP2 has SNPs associated with MG [8].

**HLA-DRB1** is ranked at position three during analysis and it plays a central role in the immune system by presenting peptides derived from extracellular proteins to helper T cells, themselves heavily implicated in EOMG [6]. It acts as a susceptibility gene in Rheumatoid arthritis [9],

**TUBB** (beta-tubulin gene) is ranked eighth in the analysis and it belongs to the family of tubulins, the major constituent of microtubules. This gene ubiquitously expresses with highest levels in spleen, thymus and immature brain. TUBB has been linked with diseases like ovarian cancer [10] and dementia [11]. [12] provided first evidence that in humans the variant form of beta1-tubulin (TUBB1 Q43P) polymorphism causes a lower reactivity in platelets and protects against thrombotic disorders but increases the risk of intracerebral hemorrhage in Spanish population. C-terminal amino acids of tubulin are recognized by Spastin (an AAA ATPase) mutated in the neurodegenerative disease hereditary spastic paraplegia) and severs microtubules [13].

**FLJ45422** is ranked 26 in the analysis and is localized at chr6: 30335353- 30342707. This gene has not been fully annotated yet and has been assigned functions such as antigen processing and presentation of peptide antigen via MHC class (http://genecards.ccbb.re.kr/cgi-bin/carddisp.pl?gene=FLJ45422). No disease has been linked with this gene. Thus, this gene could be a new potential target for association with MG as this is localized within the region highly correlated with EOMG.

Reference List


1. Fainardi E, Rizzo R, Melchiorri L, Stignani M, Castellazzi M, Tamborino C, Paolino E, Tola MR, Granieri E, Baricordi OR: **CSF levels of soluble HLA-G and Fas molecules are inversely associated to MRI evidence of disease activity in patients with relapsing remitting multiple sclerosis.** *Mult Scler* 2008.

2. Rezvany MR, Kazemi A, Hajifathali A, Kaviani S, Mellstedt H: **Analysis of HLA-G gene expression in B-lymphocytes from chronic lymphocytic leukemia patients.** *Iran Biomed J* 2007, **11:**125-129.

3. Yie SM, Yang H, Ye SR, Li K, Dong DD, Lin XM: **Expression of HLA-G is associated with prognosis in esophageal squamous cell carcinoma.** *Am J Clin Pathol* 2007, **128:**1002-1009.

4. Lajoie J, Hargrove J, Zijenah LS, Humphrey JH, Ward BJ, Roger M: **Genetic variants in nonclassical major histocompatibility complex class I human leukocyte antigen (HLA)-E and HLA-G molecules are associated with susceptibility to heterosexual acquisition of HIV-1.** *J Infect Dis* 2006, **193:**298-301.

5. Tamouza R, Busson M, Fortier C, Diagne I, Diallo D, Sloma I, Contouris H, Krishnamoorthy R, Labie D, Girot R et al.: **HLA-E*0101 allele in homozygous state favors severe bacterial infections in sickle cell anemia.** *Hum Immunol* 2007, **68:**849-853.

6. Janer M, Cowland A, Picard J, Campbell D, Pontarotti P, Newsom-Davis J, Bunce M, Welsh K, Demaine A, Wilson AG et al.: **A susceptibility region for myasthenia gravis extending into the HLA-class I sector telomeric to HLA-C.** *Hum Immunol* 1999, **60:**909-917.

7. Hodgkinson AD, Millward BA, Demaine AG: **The HLA-E locus is associated with age at onset and susceptibility to type 1 diabetes mellitus.** *Hum Immunol* 2000, **61:**290-295.

8. Hjelmstrom P, Giscombe R, Lefvert AK, Pirskanen R, Kockum I, Landin-Olsson M, Sanjeevi CB: **TAP polymorphisms in Swedish myasthenia gravis patients.** *Tissue Antigens* 1997, **49:**176-179.

9. Morel J, Roch-Bras F, Molinari N, Sany J, Eliaou JF, Combe B: **HLA-DMA*0103 and HLA-DMB*0104 alleles as novel prognostic factors in rheumatoid arthritis.** *Ann Rheum Dis* 2004, **63:**1581-1586.

10. Ohishi Y, Oda Y, Basaki Y, Kobayashi H, Wake N, Kuwano M, Tsuneyoshi M: **Expression of beta-tubulin isotypes in human primary ovarian carcinoma.** *Gynecol Oncol* 2007, **105:**586-592.

11. Revesz T, Holton JL, Doshi B, Anderton BH, Scaravilli F, Plant GT: **Cytoskeletal pathology in familial cerebral amyloid angiopathy (British type) with non-neuritic amyloid plaque formation.** *Acta Neuropathol* 1999, **97:**170-176.

12. Navarro-Nunez L, Lozano ML, Rivera J, Corral J, Roldan V, Gonzalez-Conejero R, Iniesta JA, Montaner J, Vicente V, Martinez C: **The association of the beta1-tubulin Q43P polymorphism with intracerebral hemorrhage in men.** *Haematologica* 2007, **92:**513-518.

13. White SR, Evans KJ, Lary J, Cole JL, Lauring B: **Recognition of C-terminal amino acids in tubulin by pore loops in Spastin is important for microtubule severing.** *J Cell Biol* 2007, **176:**995-1005.
